# Absence of GATA3/FOXA1 co-expression predicts poor prognosis in upper tract urothelial carcinoma

**DOI:** 10.3389/fonc.2024.1302864

**Published:** 2024-02-15

**Authors:** Yue Wang, Yunfan Wang, Huiying He, Yan Xiong

**Affiliations:** ^1^ Department of Pathology, Peking University First Hospital, Beijing, China; ^2^ Department of Pathology, Peking University Shougang Hospital, Beijing, China; ^3^ Department of Pathology, School of Basic Medical Sciences, Peking University Third Hospital, Peking University Health Science Center, Beijing, China

**Keywords:** upper tract urothelial carcinoma, prognosis, GATA3, FoxA1, necrosis

## Abstract

**Objective:**

GATA binding protein 3 (GATA3) and forkhead box A1 (FOXA1) have been individually implicated in the progression of upper tract urothelial carcinoma (UTUC). This study aims to evaluate the prognostic value of GATA3/FOXA1 co-expression in UTUC patients.

**Methods:**

We collected 108 UTUC pathological tissue samples with complete follow-up data and 24 normal control urothelial tissues. We created a 132-site microarray and performed immunohistochemistry (IHC) to measure GATA3 and FOXA1 expression levels. Kaplan-Meier survival and Cox regression analyses were conducted to assess UTUC prognosis.

**Results:**

GATA3 expression was positively correlated with FOXA1 (P=0.031). Absence of GATA3/FOXA1 co-expression (GATA3-/FOXA1-) was associated with tumor extensive necrosis (P=0.001) after Bonferroni correction for multiple comparisons. GATA3-/FOXA1- was associated with shorter Disease-Free Survival (DFS) (P=0.001) and Cancer-Specific Survival (CSS) (P<0.001) than other combination groups. Multivariate analyses identified extensive necrosis as an independent prognostic factor for CSS (P=0.030).

**Conclusions:**

Our study revealed a positive correlation between GATA3 and FOXA1 expression in UTUC. GATA3-/FOXA1- is linked to tumor extensive necrosis and poor prognosis in UTUC and may serve as a potential biomarker for UTUC patients.

## Introduction

Upper tract urothelial carcinoma (UTUC), characterized by urothelial carcinoma occurring in the renal pelvis or ureter, presents a distinct subset of urothelial tumors. While it constitutes a relatively rare entity, accounting for 5%10% of urothelial malignancies in Western countries (1), its prevalence in China is notably higher, ranging from 20% to 25% (2). Notably, UTUC patients in China exhibit unique clinical features, including advanced disease stages, existing extensive necrosis, and poorer survival outcomes, as elucidated in our prior studies (3) and supported by existing literature (4, 5). Currently, open radical nephroureterectomy (RNU) with bladder cuff excision is the standard surgical treatment for high-risk UTUC according to the European Association of Urology Guidelines (1). However, the efficacy of current therapeutic modalities is limited for many patients, emphasizing the critical need to identify novel therapeutic targets, particularly for those at the highest risk of disease progression or mortality.

GATA3, a member of the GATA family of zinc finger transcription factors, has emerged as a valuable diagnostic marker in urothelial carcinoma (6). Our previous investigations have revealed a strong association between the loss of GATA3 expression and adverse outcomes in UTUC patients (7). Mechanistically, GATA3 has been reported to function upstream of forkhead box A1 (FOXA1) (8), a member of the forkhead box A subfamily. FOXA1 has been shown to play a pivotal role in maintaining normal urothelial differentiation (9). In the context of bladder cancer, FOXA1 expression exhibits a spectrum of alterations, ranging from absence to increased or modified expression, with these anomalies associated with different molecular subtypes (10). It is of note that GATA3 and FOXA1 were co-expressed in ER-positive breast cancer and were positively correlated with clinical outcome (11). Interestingly, overexpression of GATA3 and FOXA1 can influence the expression of markers associated with the luminal molecular subtype of bladder cancer (12), suggesting potential molecular connections in UTUC. Although limited studies have reported associations between altered FOXA1 expression and poor prognosis in UTUC (13), it remains uncharted territory whether the co-expression of FOXA1 and GATA3 offers enhanced predictive value for assessing oncologic outcomes compared to either marker in isolation. This study seeks to explore the potential synergistic impact of GATA3 and FOXA1 co-expression in predicting the prognosis of UTUC patients with long-term follow-up data.

## Materials and methods

### Patients and samples

The medical records of 108 consecutive UTUC patients who underwent RNU with bladder cuff excision between January 2007 and March 2017 in Peking University Shougang Hospital were retrieved. Patients underwent biopsy only or cases without complete follow-up information were excluded. No patients received neoadjuvant chemotherapy prior to surgery. All cases were reviewed, and pathological diagnoses were confirmed independently by two pathologists. Tissue specimens were preserved in 10% buffered formalin and subsequently embedded in paraffin for pathological analysis. The histological grade was determined in accordance with the 2016 WHO Classification (14). The tumor stage was defined by the AJCC manuals. Tumor necrosis was defined as the presence of necrotic tissue exceeding 10% of the tumor area, as assessed through microscopic examination (7). Disease-Free Survival (DFS) was calculated from the date of surgery to the date when the first documented evidence of recurrent disease was noted or to the last follow-up visit for patients still alive. Cancer-Specific Survival (CSS) was defined as the time interval from the surgical procedure to death attributed to UTUC. Events were recorded for patients who died of UTUC, while those who passed away due to other causes or remained alive were censored at their last follow-up. Fourteen patients were lost to follow-up after surgery, leaving a final cohort of 94 cases for the survival analysis. The median follow-up duration was 28 months, ranging from 1 to 101 months. This retrospective study received approval from the Ethics Committee of Peking University Shougang Hospital, with a waiver of informed consent granted by patients. All research activities were conducted in compliance with relevant guidelines and regulations.

### Tissue microarray construction

TMAs were constructed from 108 samples of UTUC, and 24 samples of adjacent normal urothelium. TMAs were constructed with the three representative areas of the tumor, which were selected manually according to HE slides. The representative areas were defined as the areas with enough tumor cells, less necrosis, no extrusion and heterogeneous regions if they exist. A previous study found that ≥3 cores from each sample provides an acceptable statistical analysis in TMAs in various tumor types (15). Thus, three cores were punched from the marked area on the donor block and transferred to premade recipient paraffin block. Each core was 1 mm in diameter, and the cores were spaced 0.8 mm apart on a single glass slide.

### Immunohistochemistry staining

IHC method was chosen because it is simple and easy to perform, and thus is suitable for clinical routine practice. IHC staining was performed on 4 µm paraffin sections from the TMA. Prior to incubation with the primary antibody, the slides were deparaffinized in xylene and rehydrated in ethanol. Endogenous peroxidase activity was blocked with 3% H_2_O_2_ in PBS for 10min. Antigen retrieval was accomplished by boiling slides in Tris/EDTA buffer for 2min. Sections were incubated with primary monoclonal antibodies against GATA3 (clone L50-823; 1:200; Origene, Rockville, USA) and FOXA1 (clone 2F83; 1:100; Millipore, Temecula, CA), respectively. Following a phosphate-buffered saline wash, sections were subjected to a 30-minute incubation with secondary antibodies (Dako) at room temperature. The detection reactions utilized the Envision kit from Dako (Dako Cytomation, Glostrup, Denmark). Diaminobenzidine (DAB) was used as chromogen and hematoxylin as counterstain. Evaluation was limited to the nuclear staining of tumor cells, with any level of nuclear staining for GATA3 or FOXA1 in ≥1% of tumor cells deemed positive. Positive controls for GATA3 staining included sections from breast cancer, while prostate carcinoma sections served as positive controls for FOXA1. Negative controls, omitting the primary antibody, were generated from the same sections.

### Statistical methods

Data analysis was conducted using SPSS software (version 25.0; SPSS, Inc., Chicago, IL, USA). Categorical variables related to clinicopathological features were compared using Fishers exact test or Pearsons chi-square test. Fishers exact test was used to analyze the pairwise relationships between GATA3 and FOXA1 expression levels. Rank variables were compared using the rank-sum test. Survival analyses for CSS and DFS were performed using the KaplanMeier method and the log-rank test. Multivariate Cox regression analysis was employed for constructing predictive models with inclusion criteria based on a P-value of ≤0.1 or a risk ratio of ≤0.5 or >2 in univariate Cox analysis. All statistical tests were two-sided, and significance was set at P < 0.05. The Bonferroni correction was applied for multiple comparisons.

## Results

### General information

The characteristics of the patients are summarized in **Supplementary Table 1**. This study encompassed 108 UTUC patients. The median age at diagnosis was 70 years (ranging from 41 to 86 years), with a male-to-female ratio of 1.35:1. Among the tumors, 13.0% were categorized as low grade, while 87.0% were classified as high grade. The distribution of pathological tumor stages was as follows: pTa, 13.0%; pT1, 26.8%; pT2, 25.0%; pT3, 26.9%; and pT4, 8.3%. Accordingly, 39.8% of the tumors were characterized as non-muscle invasive, while 60.2% were deemed muscle invasive. Lympho-vascular involvement was present in 19.4% of all patients, and lymph node metastasis was observed in 9.3%. Additionally, extensive necrosis was identified in 15.7% of the tumors. During the follow-up period, complete information was available for 94 patients, among whom 28 patients succumbed to tumor progression.

### GATA3 expression in UTUC patients

**Figure 1:**
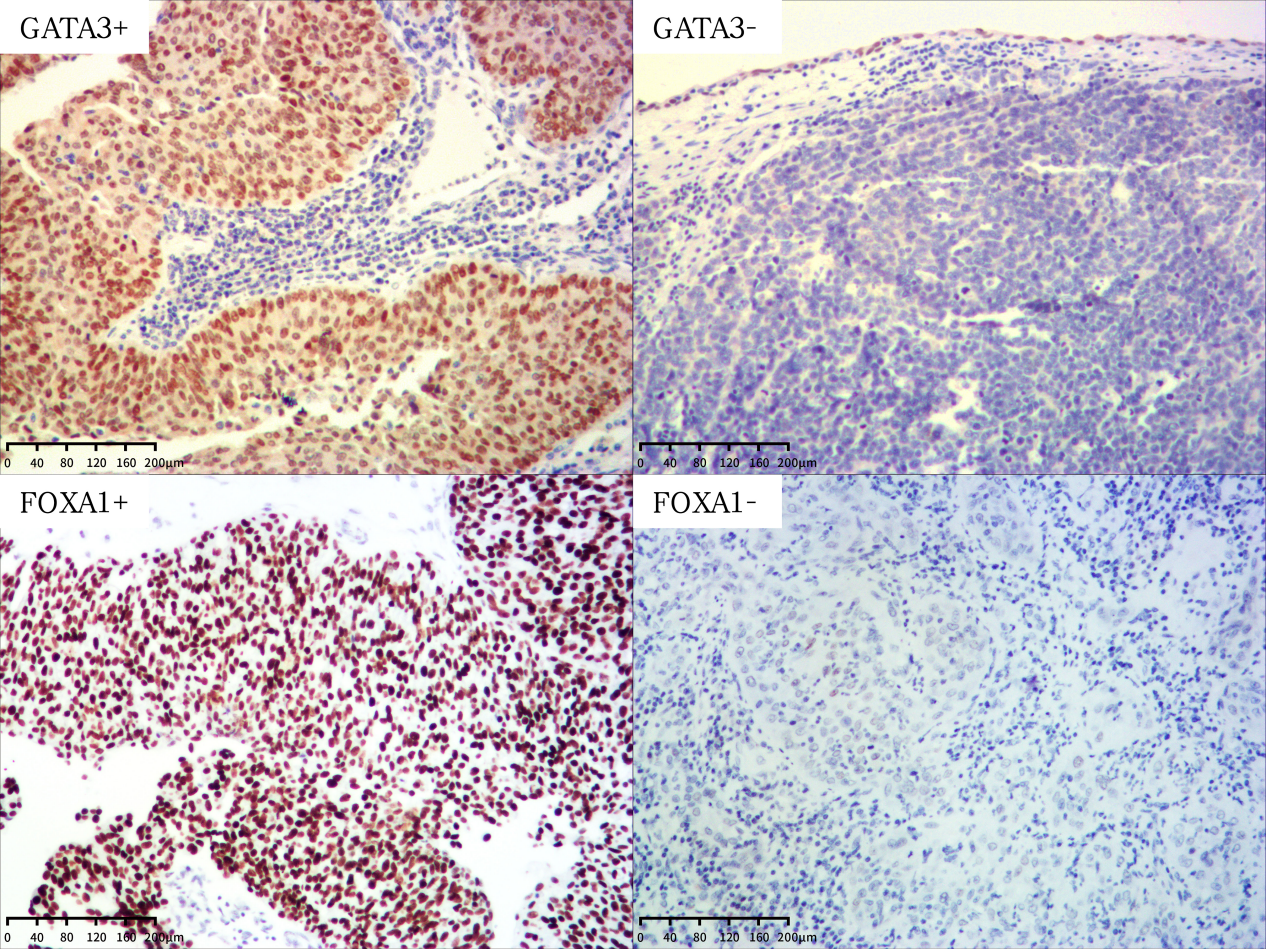
Representative images of GATA3 and FOXA1 protein expression in UTUC (magnification X100). +, positive; -, negative.

Representative images of GATA3 are depicted in **Figure 1**. We proceeded to investigate the correlation between the expression level of GATA3 and clinicopathological characteristics, as well as the prognosis of UTUC patients with long-term follow-up. The associations between GATA3 expression and clinicopathological characteristics are detailed in **Table 1**. GATA3 expression was negatively associated with pathological stage (P=0.001) and extensive necrosis (P<0.001). No additional statistical significances were detected in lympho-vascular involvement, perineural invasion, and lymph node metastasis according to multiple comparisons with Bonferroni correction. The prognostic significance of GATA3 expression is illustrated in **Supplementary Figure S1**. Negative GATA3 expression was positively correlated with shorter DFS (P=0.024; **Supplementary Figure S1A**) and shorter CSS (P=0.028; **Supplementary Figure S1B**).

**Table 1:** Association between FOXA1 and GATA3 expression and the clinicopathological features of UTUC†.

	GATA3		P*	FOXA1		P**	GATA3/ FOXA1				P***
	+	-		+	-		+/ +	+/-	-/+	-/-	
Patient age											
< 65	29	11	0.528	37	3	0.708	28	9	1	2	0.868
≥ 65	45	23		64	4		44	20	1	3	
Sex											
Male	43	19	0.837	58	4	1.000	42	16	1	3	0.970
Female	31	15		43	3		30	13	1	2	
Laterality											
Left	35	14	0.358	47	2	0.485	34	13	1	1	0.489
Right	39	19		53	5		38	15	1	4	
Both	0	1		1	0		0	1	0	0	
Tumor site											
Renal pelvis	28	14	0.048	37	4	0.537	27	11	1	3	0.174
Ureter	42	13		52	3		41	11	1	2	
Transitional zone	4	6		10	0		4	6	0	0	
Renal pelvis & Ureter	0	1		1	0		0	1	0	0	
Tumor size											
< 3.0cm	44	16	0.298	57	3	0.698	43	14	1	2	0.656
≥ 3.0cm	30	18		44	4		29	15	1	3	
Tumor grade											
Low grade	10	4	1.000	31	0	0.189	24	7	0	0	0.378
High grade	64	30		70	7		48	22	2	5	
Pathological stage											
NMI (pTa-pT1)	37	6	**0.001**	43	0	0.040	36	7	0	0	0.009
MI (pT2-pT4)	37	28		58	7		36	22	2	5	
LVI											
No	64	23	0.035	82	5	0.620	62	20	2	3	0.109
Yes	10	11		19	2		10	9	0	2	
Perineural invasion											
No	72	27	0.004	92	7	1.000	70	22	2	5	0.010
Yes	2	7		9	0		2	7	0	0	
Lymph node metastasis											
No	71	27	0.010	92	6	0.504	69	23	2	4	0.041
Yes	3	7		9	1		3	6	0	1	
Concurrent CIS											
No	62	31	0.381	87	6	1.000	60	27	2	4	0.542
Yes	12	3		14	1		12	2	0	1	
Extensive necrosis											
No	69	22	**<0.001**	87	4	0.076	67	20	2	2	**0.001**
Yes	5	12		14	3		5	9	0	3	
Glomerular sclerosis											
No	53	20	0.268	67	6	0.424	51	16	2	4	0.350
Yes	21	14		34	1		21	13	0	1	
Solitary or Multifocal											
Solitary	65	28	0.550	86	7	0.590	63	23	2	5	0.588
Multifocal	9	6		15	0		9	6	0	0	
Bladder cancer^a^											
No	46	15	0.653	58	3	1.000	45	13	1	2	0.880
Simultaneous	4	1		5	0		4	1	0	0	
Postoperative	10	6		15	1		10	5	0	1	
Preoperative	2	0		2	0		2	0	0	0	

^†^Threshold levels of significance were adjusted for multiple comparisons by Bonferroni correction. The adjusted alpha value was 0.0011 (0.05/45). Bold values meant statistically significant P-values after Bonferroni correction.

P*: comparison between the GATA3+ group and the GATA3- group.

P**: comparison between the FOXA1+ group and the FOXA1- group.

P***: comparison among GATA3+/FOXA1+, GATA3+/FOXA1-, GATA3-/FOXA1+, and GATA3-/FOXA1- groups.

UTUC, upper tract urothelial carcinoma; NMI, non-muscle invasive; MI, muscle invasive; LVI, Lympho-vascular involvement; CIS, carcinoma in situ; +, positive; -, negative.

^a^Clinical information on bladder cancer recurrence was obtained in 84 patients.

### FOXA1 expression in UTUC patients

Subsequently, we examined the association between FOXA1 expression and clinicopathological characteristics, as well as the prognosis of UTUC patients. This analysis was conducted using the same TMAs employed for the expression analysis of GATA3. Representative images of FOXA1 are presented in **Figure 1**. The associations between FOXA1 expression and clinicopathological characteristics are outlined in **Table 1**. No statistically significance was found after the Bonferroni correction for multiple comparisons. The prognostic relevance of FOXA1 expression is depicted in **Supplementary Figure S1**. Negative FOXA1 expression was positively associated with unfavorable prognosis in both DFS (P<0.001; **Supplementary Figure S1C**) and CSS (P<0.001; **Supplementary Figure S1D**).

### Combination of GATA3 and FOXA1 expression in UTUC patients

**Figure 2:**
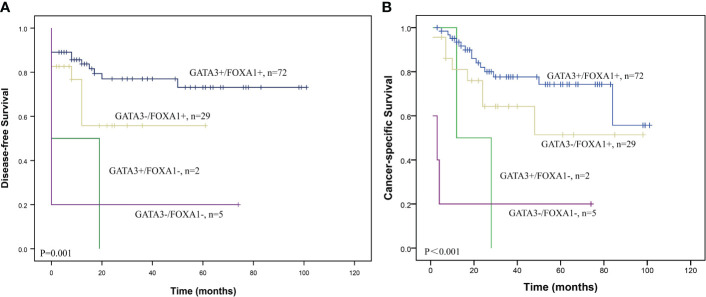
Survival curves according to the combination of GATA3 and FOXA1 protein expression in UTUC. Graphs show **(A)** DFS and **(B)** CSS curves.

We then assessed the clinicopathological value and prognostic impact of the combined expression of GATA3 and FOXA1. A majority of GATA3 positive cases also exhibited positive FOXA1 expression (97.3%), and there was a positive correlation between the expression levels of GATA3 and FOXA1 (P=0.031; **Supplementary Table 2**). Patients were categorized into four groups based on the expression patterns of GATA3 and FOXA1 proteins: GATA3+/FOXA1+ (n=72), GATA3+/FOXA1- (n=29), GATA3-/FOXA1+ (n=2), and GATA3-/FOXA1- (n=5). The co-expression of GATA3 and FOXA1 was solely adversely associated with the existing of extensive necrosis (P=0.001) according to multiple comparisons with Bonferroni correction (**Table 1**). No additional statistical significances were found in pT stage, perineural invasion, and lymph node metastasis. Among the above four GATA3/FOXA1 groups, the GATA3+/FOXA1+ group exhibited the longest DFS and CSS, with statistically significant differences (P=0.001 for DFS, P<0.001 for CSS; **Figure 2**). Then we pairwise compared survival differences between every two groups. As a result, there were three pairs reached statistical significance, that is GATA3+/FOXA1+ group vs. GATA3+/FOXA1- group (P=0.006 for DFS, P=0.004 for CSS), GATA3+/FOXA1+ group vs. GATA3-/FOXA1- group (P=0.001 for DFS, P<0.001 for CSS), and GATA3-/FOXA1+ group vs. GATA3-/FOXA1- group (P=0.033 for DFS, P=0.007 for CSS) (**Supplementary Figure S2**).

Next, we conducted univariate and multivariate analyses of prognostic variables for UTUC. In addition to the GATA3/FOXA1 combination expression, tumor grade, pT stage, lympho-vascular involvement, lymph node metastasis, and extensive necrosis all contributed to DFS and CSS in the univariate analysis (**Table 2**). Factors that demonstrated significance in the univariate analysis were subsequently subjected to multivariate Cox proportional hazards analysis. However, the combination of GATA3/FOXA1 did not emerge as an independent prognostic marker for either DFS or CSS. Among all clinicopathological features, only extensive necrosis exhibited independent prognostic value in CSS (**Table 2**).

**Table 2:** Univariate and multivariate analysis of disease-free survival and cancer-specific survival in patients with UTUC.

	Disease-free Survival						Cancer-specific Survival					
	Univariate			Multivariate			Univariate			Multivariate		
	HR	95%CI	*P*	HR	95%CI	*P*	HR	95%CI	*P*	HR	95%CI	*P*
All cases (n = 108)												
GATA3/FOXA1	1.583	1.137-2.203	**0.001**	1.136	0.784-1.646	0.500	1.682	1.193-2.371	**<0.001**	1.274	0.862-1.884	0.224
Tumor Grade	35.464	1.149-1.094E3	**0.041**	0.974	0.000-1.507E137	1.000	36.575	1.262-1.060E3	**0.036**	1.034	0.000-7.442E113	1.000
pT stage	48.004	2.107-1.093E3	**0.015**	70315.192	0.000-1.608E122	0.935	50.838	2.309-1.119E3	**0.013**	43499.385	0.000-2.193E102	0.926
LVI	2.345	1.082-5.086	**0.031**	1.262	0.535-2.973	0.595	2.379	1.089-5.200	**0.030**	1.295	0.551-3.043	0.553
Perineural invasion	1.785	0.617-5.165	0.285	0.925	0.288-2.968	0.896	1.994	0.687-5.787	0.204	0.923	0.283-3.005	0.894
LN metastasis	3.793	1.422-10.123	**0.008**	1.293	0.440-3.800	0.640	4.734	1.751-12.802	**0.002**	1.600	0.515-4.969	0.416
Concurrent CIS	0.882	0.306-2.545	0.816	0.712	0.229-2.216	0.557	0.883	0.305-2.554	0.818	0.859	0.267-2.762	0.799
Extensive necrosis	5.410	2.424-12.074	**<0.001**	2.578	0.986-6.739	0.053	6.288	2.864-13.804	**<0.001**	2.750	1.102-6.864	**0.030**

UTUC, upper tract urothelial carcinoma; LVI, Lympho-vascular involvement; LN, lymph node; CIS, carcinoma in situ.

Bold values meant P<0.05.

## Discussion

In this study, we primarily focused on evaluating the prognostic value of the co-expression of GATA3 and FOXA1 in UTUC patients with long-term follow-up. Our findings revealed several key insights as follows.

First is correlation between GATA3 and FOXA1 expression. We demonstrated a positive correlation between the expression levels of GATA3 and FOXA1 in UTUC. This is in line with previous researches in breast cancer, where GATA3/FOXA1 co-expressed in ER-positive cancers (11), as well as *GATA3* and *FOXA1* genes were the most upregulated out of their corresponding transcription factors in BRCA cancer samples (16). Mechanically, GATA3 was shown to function upstream of FOXA1 (8). A recent study observed a gain of DNA methylation at direct GATA3 or FOXA1 binding sites, and validated these two transcription factors mediate hypo-methylated regions, thus resulting in shaping DNA methylation patterns in breast cancer (16). While previous studies exploring the role of GATA3/FOXA1 focused mostly on breast cancer, there were limited studies on urothelial carcinoma, especially UTUC. One research on bladder cancer found that overexpression of GATA3 and FOXA1 could influence the expression of markers associated with the luminal molecular subtype (12), suggesting potential molecular connections in UTUC. Further studies are needed to dive deeper into the potential mechanisms by which GATA3 and FOXA1 expressions are altered in UTUC.

Second is clinicopathological associations. The absence of GATA3/FOXA1 expression was solely significantly associated with the existing of extensive necrosis (P=0.001) according to multiple comparisons with Bonferroni correction. To the best of our knowledge, there was only one study which included the IHC expression levels of GATA3 and FOXA1, as well as the prognostic value of extensive necrosis in bladder cancer (17). However, they didnt show the correlation between IHC results and extensive necrosis. In the context of bladder cancer, the FOXA1 expression can be altered according to different molecular subtypes (18), with elevating in luminal molecular subtype whereas diminishing in basal molecular subtype, which is in keeping with the pattern of FOXA1 expression during urothelial differentiation. Thus, additional effort is required to identify molecular subtypes of UTUC to further verify these findings.

Third is prognostic significance. Most GATA3/FOXA1 studies, mostly focusing on breast cancer, demonstrated that the combination of the two indicators can better predict the prognosis than either indicator alone (19, 20). Patients with absent GATA3 and FOXA1 expression (GATA3-/FOXA1-) exhibited the shortest survival time compared to other combination groups in our study. This suggests that the co-expression of these markers may have stratification value and prognostic significance in UTUC, although it was not an independent predictor of outcome in multivariate analysis. As there were few cases in the GATA3-/FOXA1+ and GATA3-/FOXA1- groups, further studies with larger cohorts are needed to validate these findings. Previous studies have shown that GATA3 expression is associated with a better prognosis in UTUC (7, 21). Our study extended this by investigating FOXA1 expression and its relationship with prognosis. The prognostic significance of the FOXA1 expression in urothelial carcinoma varies among different studies. Generally, most studies demonstrated that decreased FOXA1 expression correlated with poor oncological outcomes (18). However, there were a few studies that hold different perspectives. Sikic et al. showed that high expression of FOXA1was associated with reduced survival time in muscle-invasive bladder cancer (22). He et al. found that increased FOXA1 expression promoted cancer immuno- and chemotherapy resistance in bladder cancer patients (23). In UTUC, our findings were consistent with most studies, indicating that lower FOXA1 expression correlated with an unfavorable prognosis. However, more research is needed to understand the role of FOXA1 in UTUC and its potential association with different molecular subtypes.

Finally, its important to acknowledge the limitations of this study, including its retrospective nature, relatively small sample size, and reliance on immunohistochemical analysis. Larger-scale prospective studies, as well as cellular research, are warranted to further validate the prognostic significance of GATA3 and FOXA1 co-expression in UTUC. Additionally, future research should aim to uncover the underlying mechanisms by which GATA3 and FOXA1 expression is altered in UTUC and how these alterations contribute to adverse clinical outcomes.

In summary, while this study provides valuable insights into the potential prognostic significance and stratification value of GATA3/FOXA1 combination in UTUC, further research is needed to confirm and expand upon these findings. Understanding the molecular mechanisms underlying these markers in UTUC could potentially lead to improved diagnostic and targeted therapeutic strategies for this disease.

## Conclusion

In conclusion, our study revealed that the absence of GATA3/FOXA1 expression is linked to tumor extensive necrosis and poor prognosis in UTUC. Although absent GATA3/FOXA1 expression was not an independent prognostic factor, the finding that patients with GATA3-/FOXA1- exhibited the shortest survival highlights the prognostic significance and stratification value of this combination. These results suggest that the GATA3-FOXA1 axis may have therapeutic implications in UTUC patients. As there were several limitations of our study due to few cases in GATA3-/FOXA1+ and GATA3-/FOXA1- groups, as well as limitations due to the fact that population tested was just Asiatic population from one single institution, a multicenter study with larger cohorts is expected. Further research is needed to validate these findings and explore the underlying molecular mechanisms, ultimately paving the way for potential targeted therapies in UTUC management.

## Data availability statement

The raw data supporting the conclusions of this article will be made available by the authors, without undue reservation.

## Ethics statement

The present study was performed according to the principles of the Helsinki declaration. Experimental protocols were reviewed and approved by the Ethics Committee of the Peking University Shougang Hospital (approval no. IRBK‑2017‑047‑01).

## Author contributions

YueW: Conceptualization, Writing – original draft. YunW: Methodology, Writing – original draft. HH: Conceptualization, Writing – review & editing. YX: Writing – review & editing.

## Supplementary material

Supplementary Figure 1 Survival curves for GATA3 and FOXA1 expression in UTUC. Graphs show DFS and CSS curves for GATA3 expression **(A, B)**, as well as DFS and CSS curves for FOXA1 expression **(C, D)**.

Supplementary Figure 2 Pairwise comparison of survival curves for GATA3/FOXA1 expression in UTUC. **(A)** DFS and **(B)** CSS survival differences between the GATA3+/FOXA1+ group and the GATA3+/FOXA1- group. **(C)** DFS and **(D)** CSS survival differences between the GATA3+/FOXA1+ group and the GATA3-/FOXA1- group. **(E)** DFS and (f) CSS survival differences between the GATA3-/FOXA1+ group and the GATA3-/FOXA1- group.
