# Supplementary figures and images for: Absence of GATA3/FOXA1 co-expression predicts poor prognosis in upper tract urothelial carcinoma
Source: Front Oncol. 2024 Feb 15;14:1302864. doi: 10.3389/fonc.2024.1302864 (PMC10902436; doi:10.3389/fonc.2024.1302864)

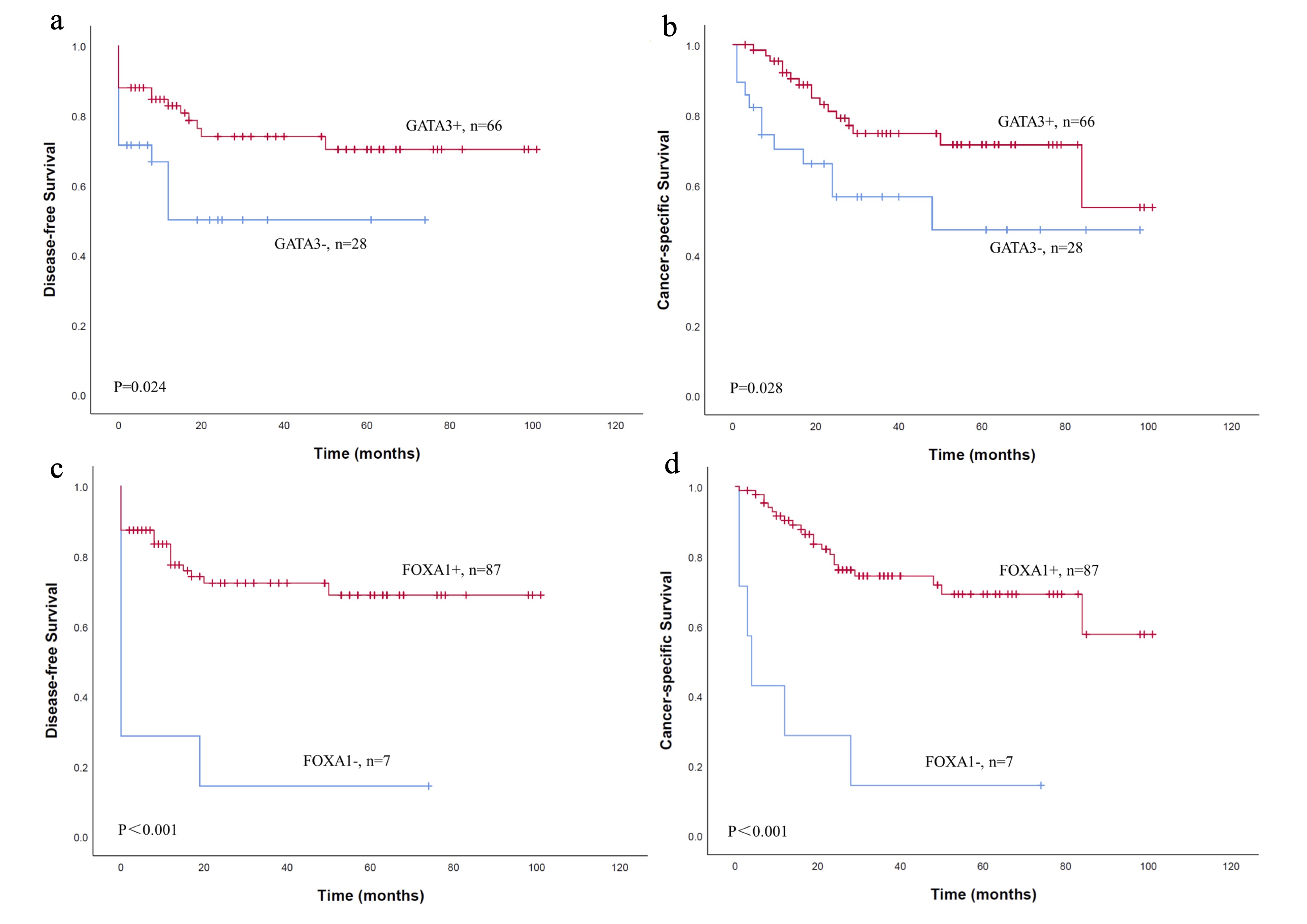

Supplement: Supplementary Figure 1 — Survival curves for GATA3 and FOXA1 expression in UTUC. Graphs show DFS and CSS curves for GATA3 expression (A, B), as well as DFS and CSS curves for FOXA1 expression (C, D). [file Image_1.jpeg]

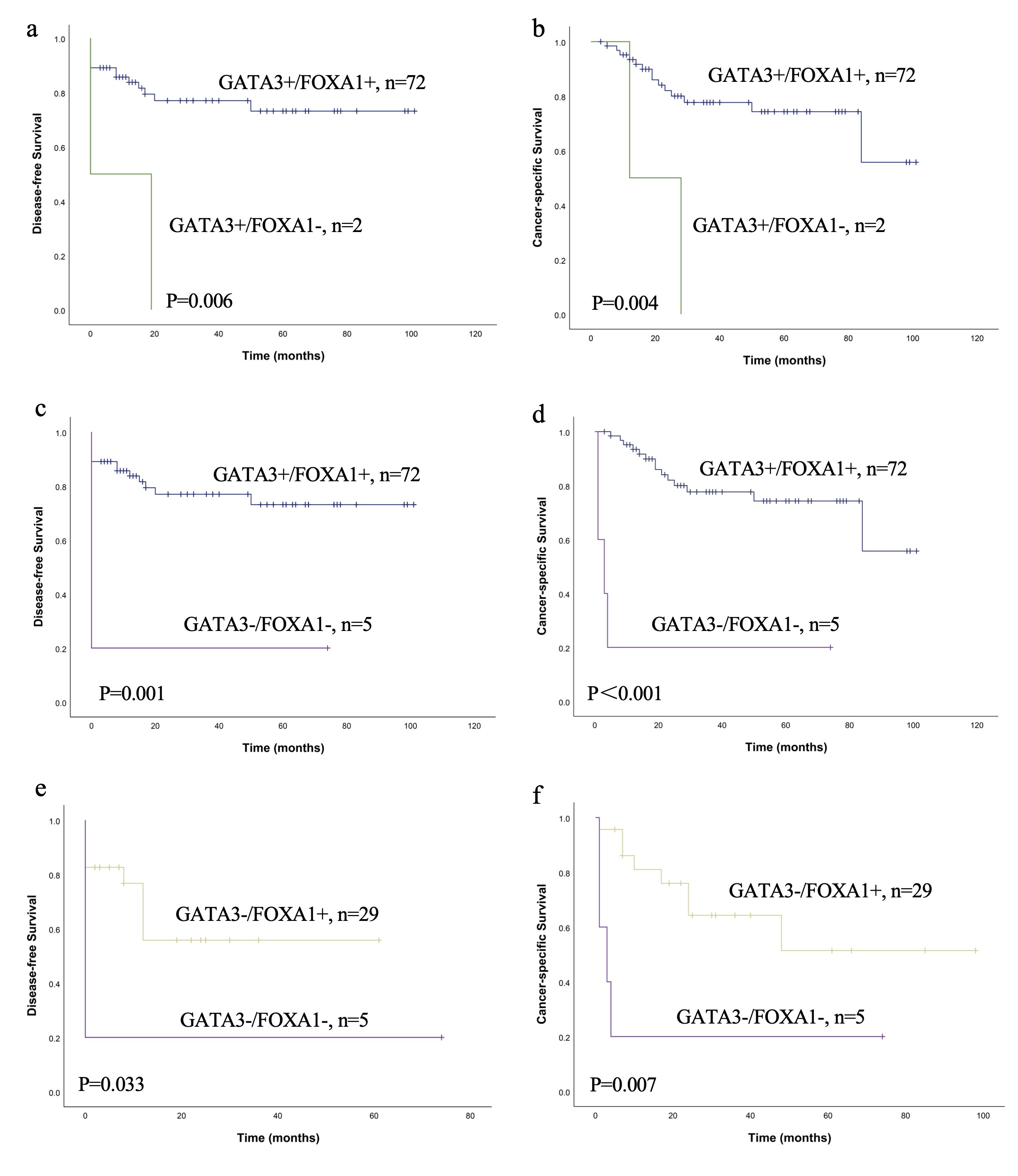

Supplement: Supplementary Figure 2 — Pairwise comparison of survival curves for GATA3/FOXA1 expression in UTUC. (A) DFS and (B) CSS survival differences between the GATA3+/FOXA1+ group and the GATA3+/FOXA1- group. (C) DFS and (D) CSS survival differences between the GATA3+/FOXA1+ group and the GATA3-/FOXA1- group. (E) DFS and (f) CSS survival differences between the GATA3-/FOXA1+ group and the GATA3-/FOXA1- group. [file Image_2.jpeg]
