# Supplementary material for: Absence of GATA3/FOXA1 co-expression predicts poor prognosis in upper tract urothelial carcinoma
Source: Front Oncol. 2024 Feb 15;14:1302864. doi: 10.3389/fonc.2024.1302864 (PMC10902436; doi:10.3389/fonc.2024.1302864)
Supplement: Supplementary file 3 [file Table_1.docx]

**Supplementary Table 1** Patient characteristics at diagnosis

| Variable | *n* | % | Variable | *n* | % |
| --- | --- | --- | --- | --- | --- |
| Patient age |  |  | Lymph node metastasis |  |  |
| < 65 | 40 | 37.0 | No | 98 | 90.7 |
| ≥ 65 | 68 | 63.0 | Yes | 10 | 9.3 |
| Sex |  |  | Concurrent CIS |  |  |
| Male | 62 | 57.4 | No | 93 | 86.1 |
| Female | 46 | 42.6 | Yes | 15 | 13.9 |
| Laterality |  |  | Extensive necrosis |  |  |
| Left | 49 | 45.4 | No | 91 | 84.3 |
| Right | 58 | 53.7 | Yes | 17 | 15.7 |
| Both | 1 | 0.9 | Glomerular sclerosis |  |  |
| Tumor site |  |  | No | 73 | 67.6 |
| Renal pelvis | 42 | 38.9 | Yes | 35 | 32.4 |
| Ureter | 55 | 50.9 | Solitary or Multifocal |  |  |
| Transitional zone | 10 | 9.3 | Solitary | 93 | 86.1 |
| Renal pelvis& Ureter | 1 | 0.9 | Multifocal | 15 | 13.9 |
| Tumor size |  |  | Bladder cancer |  |  |
| < 3.0cm | 60 | 55.6 | No | 71 | 75.6 |
| ≥ 3.0cm | 48 | 44.4 | Simultaneous | 5 | 5.3 |
| Tumor grade |  |  | Postoperative | 16 | 17.0 |
| Low grade | 14 | 13.0 | Preoperative | 2 | 2.1 |
| High grade | 94 | 87.0 | Median follow-up time (month) | 28 |  |
| Pathological stage |  |  | Status |  |  |
| pTa | 14 | 13.0 | Survival | 66 | 70.2 |
| pT1 | 29 | 26.8 | Death | 28 | 29.8 |
| NMI (pTa + pT1) | 43 | 39.8 | Disease-free survival |  | 67.5% ^a^ |
| pT2 | 27 | 25.0 | < 1 year | 36 |  |
| pT3 | 29 | 26.9 | 1‒3 years | 31 |  |
| pT4 | 9 | 8.3 | > 3 years | 27 |  |
| MI (pT2 + pT3 + pT4) | 65 | 60.2 | Cancer-specific survival |  | 69.4% ^b^ |
| Lympho-vascular involvement |  |  | < 1 year | 19 |  |
| No | 87 | 80.6 | 1‒3 years | 41 |  |
| Yes | 21 | 19.4 | > 3 years | 34 |  |
| Neural invasion |  |  |  |  |  |
| No | 99 | 91.7 |  |  |  |
| Yes | 9 | 8.3 |  |  |  |

Abbreviations: NMI, non-muscle invasive; MI, muscle invasive; CIS, carcinoma in situ.

^a^ The 3-year Disease-free survival rate of UTUC. ^b^ The 3-year Cancer-specific survival rate of UTUC.

**Supplementary Table 2** Correlation between GATA3 and FOXA1 expression

|  |  | FOXA1 | | P |
| --- | --- | --- | --- | --- |
|  |  | +(n) | -(n) |  |
| GATA3 | +(n) | 72 | 2 | 0.031 |
|  | -(n) | 29 | 5 |  |
